# 1.5 Tesla Non-ultrashort but Short Echo Time Magnetic Resonance Angiography Describes the Arteries Near a Clipped Cerebral Aneurysm

**DOI:** 10.7759/cureus.16611

**Published:** 2021-07-25

**Authors:** Yoichi Higo, Sakura Komagata, Masahito Katsuki, Shin Kawamura, Akihito Koh

**Affiliations:** 1 Department of Radiological Technology, Itoigawa General Hospital, Itoigawa, JPN; 2 Department of Neurosurgery, Itoigawa General Hospital, Itoigawa, JPN

**Keywords:** cerebral aneurysm, clipping, time-of-flight magnetic resonance angiography (tof-mra), ultrashort echo time magnetic resonance angiography (ute-mra), less invasive, magnetic resonance imaging, clip artifact

## Abstract

Cerebral aneurysm and mother artery assessment after clipping is essential to evaluate aneurysm remnant, regrowth, and clip slippage. Usually, cerebral angiography and contrast-enhanced computed tomography angiography (CTA) are used for the evaluation, but they have the side effect of contrast medium and are time-consuming. Time-of-flight magnetic resonance angiography (TOF-MRA) is a non-invasive and fast modality, but clip-induced artifacts limit the signal near the metal clip. Recent ultrashort echo time (UTE)-MRA reduces metal artifacts but its availability is still low worldwide. Therefore, we developed a modified TOF-MRA sequence, named short TE-MRA, using Optima MR 360 1.5T Advance (GE Healthcare Life Sciences, Buckinghamshire, UK). It could describe the artery near the clip using general MRA equipment without recent UTE-MRA technology. We present a subarachnoid hemorrhage patient who underwent short TE-MRA about a year after clipping for the aneurysms at the bilateral internal carotid arteries. Short TE-MRA described the left internal carotid, middle cerebral, and anterior cerebral arteries. The right middle and anterior cerebral arteries were described, but the right internal carotid artery was not. Normal TOF-MRA could not describe them. Without recent UTE-MRA technology, short TE-MRA might be an alternative method for evaluating the artery near the clip. Short TE-MRA can be performed by general MRA equipment with a little time, so it may be helpful until UTE-MRA is widely used. Further research is needed on whether short TE-MRA can describe the aneurysm remnant, regrowth, and clip slippage up to the clinically useful level.

## Introduction

The cerebral aneurysm and mother artery assessment after clipping is essential to evaluate aneurysm remnant, regrowth, clip slippage, and kink of the arteries. Usually, cerebral angiography (CAG) and contrast-enhanced computed tomography angiography (CTA) are used for the evaluation [1], but they have side effects of contrast medium and are time-consuming. Of course, less invasive methods are better for the aneurysm and artery assessment. Time-of-flight magnetic resonance angiography (TOF-MRA) is a non-invasive and fast modality, but clip-induced metal artifact limits the signal near the clip [2]. Recent ultrashort echo time (UTE)-MRA reduces metal artifact [3-11]. However, UTE-MRA is a recent sequence and costs much, so its availability is still low worldwide. Therefore, we tried to make a new sequence, which describes the artery near the metal clip without the latest MRA equipment. We developed a modified TOF-MRA sequence, named short TE-MRA, using Optima MR 360 1.5T Advance (GE Healthcare Life Sciences, Buckinghamshire, UK). Short TE-MRA could describe the artery near the clip without recent UTE-MRA technology nor other modalities using contrast medium.

## Case presentation

**Figure 1:**
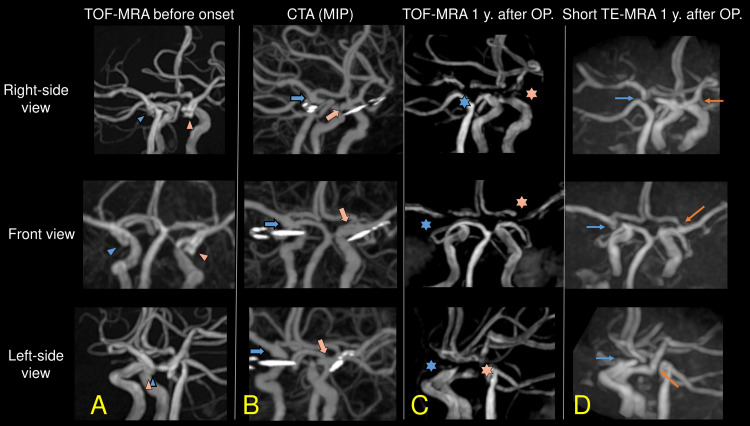
Comparison of each MRA sequence TOF-MRA before the subarachnoid hemorrhage onset revealed the 7 mm aneurysm at the left ICA (red arrowheads, A) and the 5 mm aneurysm at the right ICA (blue arrowheads; A). On the third postoperative day after clipping, CTA revealed the clips and bilateral ICAs (thick red and blue arrows; B). About a year after the clipping, 1.5T TOF-MRA did not describe the bilateral ICAs, nor some parts of MCAs and ACAs (red and blue asterisks; C). Short TE-MRA did not describe the right ICA (thin blue arrows, D), but it revealed the right MCA and A1 portion of the ACA. Also, it revealed those on the left (thin red arrows, D). Abbreviations in the figures: CTA; computed tomography angiography, OP; operation, MIP; maximum intensity projection, TE; echo time TOF-MRA; time-of-flight magnetic resonance angiography

An 80-year-old woman developed subarachnoid hemorrhage (SAH), Hunt and Kosnik grade II, due to a ruptured aneurysm at the left internal carotid artery (ICA). She also had an unruptured aneurysm at the right ICA. TOF-MRA five years before the onset revealed the 7 mm aneurysm at the left ICA (red arrowheads, Figure 1, panel A) and the 5 mm aneurysm at the right ICA (blue arrowheads, Figure 1, panel A). We performed first left and then right frontotemporal craniotomies and clipping using the fourth-generation YASARGIL titanium clips #760 (Aesculap, Tuttlingen, Germany) as a two-stage surgery in three days, just in case of misidentifying the ruptured aneurysm [12]. The clips were made of TiAl6V4 titanium alloy (ISO 5832-2). On the three-postoperative day after the second surgery, CTA revealed the clips and bilateral ICAs, and complete clipping was confirmed (thick red and blue arrows, Figure 1, panel B). The patient was discharged home after a two-month rehabilitation with a modified Rankin Scale 4.

About a year after the surgery, we performed TOF-MRA and short TE-MRA to evaluate the bilateral arteries near the clip. 1.5T TOF-MRA did not describe the bilateral ICAs. Furthermore, some parts of the bilateral middle cerebral arteries (MCAs) and A1 portions of the anterior cerebral arteries (ACAs) were not described (red and blue asterisks, Figure 1, panel C). Short TE-MRA did not describe the right ICA, but it revealed the right MCA and A1 portion of the ACA (thin blue arrows, Figure 1, panel D). Short TE-MRA revealed those on the left (thin red arrows, Figure 1, panel D). The bilateral posterior communicating arteries were not described in both CTA and short TE-MRA due to their narrow diameters and the metal artifacts of the clips.

We acquired the CTA using an 80-row CT scanner (Aquillion Prime SP; Canon Medical Systems, Tochigi, Japan). Scan parameters comprised rotation period, 0.5 sec; tube voltage, 20 kV; tube current, 150-350 mA; slice thickness, 0.5 mm; matrix, 512 × 512; and field of view, 200 mm. Iopamidol was used as a contrast medium. The maximum intensity projection images were made using Aquarius iNtuition Client Viewer (TeraRecon Incorporated, Durham).

To acquire the TOF-MRA and short TE-MRA, we used Optima MR 360 1.5T Advance with an 8-channel head-neck coil. The detailed scan parameters for TOF-MRA: repetition time/echo time, 32/6.8 ms; flip angle, 18 degrees; field of view, 200 × 200 mm; matrix, 288 × 192; thickness, 1.2 mm; and acquisition time, 301 seconds. Those for short TE-MRA: repetition time/echo time, 32/1.2 ms; flip angle, 18 degrees; field of view, 220 × 220 mm; matrix, 160 × 160; thickness, 1.6 mm; and acquisition time, 173 seconds. The images of TOF- and short TE-MRA were acquired as axial images.

Our hospital’s research ethics committee approved this study (ethical approval number 2021-4). We gained written informed consent for this study from the patients. All methods were carried out under relevant guidelines and regulations (Declaration of Helsinki).

## Discussion

This is the first report on the clinical usefulness of modified TOF-MRA, named short TE-MRA, for post-clipping assessment on the cerebral arteries. Compared to CTA, short TE-MRA is less invasive and fast, but its image quality is not up to the CTA. The description range and the image quality of short TE-MRA were inferior to TOF-MRA, but short-TE MRA could describe some of the mother vessels. Compared to UTE-MRA, short TE-MRA does not need recent special equipment for UTE-MRA [13], so short TE-MRA is available using standard equipment worldwide. Furthermore, the acquisition time for UTE-MRA is long as five to 10 minutes due to multiple scans and subtraction processing [13]. In contrast, the acquisition time for short TE-MRA is only 173 seconds, so it is not a labor-intensive and time-consuming task to be incorporated into daily magnetic resonance imaging scans.

We hypothesize that UTE-MRA, such as pointwise encoding time reduction with radial acquisition (PETRA)-MRA (Siemens, Germany) [8] and SILENT MRA (GE Healthcare Life Sciences, UK) [9], will be the gold standard to evaluate the aneurysms and arteries after clipping in the future as to CAG and CTA. However, they have not yet been widespread. Of course, CAG and CTA are superior to both UTE-MRA and short TE-MRA for the description of the arteries near the clip [14]. However, short TE-MRA is less invasive and not time-consuming so it may be helpful for routine outpatient follow-up for the patients after clipping instead of frequent CAG or CTA.

## Conclusions

Short TE-MRA could be performed by general MRA equipment, and it takes a little time. We performed the sequence without the latest MRA equipment. It described an artery that the usual TOF-MRA could not show. Without a recent UTE-MRA sequence, short TE-MRA might be an alternative method for evaluating the artery near the clip. Further research is needed on whether short TE-MRA can describe the aneurysm remnant, regrowth, and clip slippage. Also, it should be clarified what kind of aneurysm, clip shape, and surgical procedure, like how to apply the clip, can be applied to short TE-MRA.
